# High-Performance Flexible Piezoresistive Sensor Based on Ti_3_C_2_T_x_ MXene with a Honeycomb-like Structure for Human Activity Monitoring

**DOI:** 10.3390/mi13060821

**Published:** 2022-05-25

**Authors:** Yue Su, Kainan Ma, Fang Yuan, Jun Tang, Ming Liu, Xu Zhang

**Affiliations:** Institute of Semiconductors, Chinese Academy of Sciences, Beijing 100864, China; yuesu@semi.ac.cn (Y.S.); makainan@semi.ac.cn (K.M.); fyuan@semi.ac.cn (F.Y.); junt@semi.ac.cn (J.T.)

**Keywords:** flexible piezoresistive sensor, MXene, honeycomb-like structure, femtosecond filamentating pulses

## Abstract

Wearable and flexible pressure sensors have sparked great interest due to their unique capacity to conformally attach to the surface of the skin and quantify human activities into recordable electric signals. As a result, more and more research efforts are being devoted to developing high-sensitivity and cost-effective flexible sensors for monitoring an individual’s state of activity. Herein, a high-performance flexible piezoresistive sensor was designed and fabricated by combing 2D transition metal carbides, nitrides, and carbonitrides (MXene) with a honeycomb-like structure formed by femtosecond filamentating pulses. The sensing mechanism is attributed to the change of the connecting conductive paths between the top interdigital electrodes and the bottom microstructured films coated with MXene. The obtained sensing device demonstrates high sensitivity of 0.61 kPa^−1^, relatively short response time, and excellent reliability and stability. Benefiting from the aforementioned extraordinary sensing performance, the sensor can be used with success to monitor tiny physiological signals, detect large deformations during human movement, and distinguish finger gestures, thus demonstrating its broad prospects in physiological analysis systems, health monitoring systems, and human–machine interaction.

## 1. Introduction

Flexible pressure sensors with excellent force-to-electric ability have aroused a huge surge of research interest due to their enormous potential in diverse applications, such as personal healthcare monitoring, artificial intelligence interaction, humanoid robotics, etc. [1,2,3,4,5]. Over the years, great efforts have been devoted to developing high-performance sensing devices to capture information feedback from various human activities, which is of great value for timely diagnosis and treatment of the disease [6]. Compared with capacitive [7,8], piezoelectric [9,10,11], and triboelectric [12] pressure sensors, piezoresistive-type sensors have been considered excellent candidates for next-generation sensors due to their merits of remarkable sensing performance, feasible manufacturing technique, excellent conformal contact, and low energy consumption [6,13,14]. Despite considerable advances recently, the low-cost, large-scale fabrication of piezoresistive pressure sensors integrating low-strain and large deformation detecting capabilities still faces enormous challenges.

Rational selection of sensing materials has been employed as an important factor to achieve the fabrication of high-performance and low-cost flexible pressure sensors. Various conductive materials, such as graphene [15], conductive polymer [16], metal nanowires [17], and carbon nanotubes (CNTs) [18], have been demonstrated as the active layers for flexible piezoresistive sensors due to their great conductivity and excellent mechanical properties. In addition, two-dimensional transition metal dichalcogenides such as MoSe_2_ nanosheets [19] and WSe_2_ nanosheets [20] can also be used as active material. The emerging two-dimensional transition metal carbides, nitrides, and carbonitrides (MXenes), based on their characteristics of adjustable interlayer spacing, excellent mechanical flexibility, and outstanding metallic conductivity, have great potential for developing next-generation high-performance piezoresistive sensors [21,22,23]. MXenes are typically obtained by selectively etching the A-layer from a ceramic material called the MAX phase and can be defined as $M_{n+1}X_{n}T_{x}$ (n = 1, 2, 3), where M denotes an early transition metal, X represents carbon or nitrogen, and T means surface functional groups [24]. In recent years, research on MXenes-based pressure sensors has been gradually carried out. Unfortunately, MXene-based sensors rapidly reach the deformation limit under external pressure due to the single 2D planar structure of MXene materials [25] and the unavoidable stacking during the preparation process [26], thereby limiting device performance. Therefore, there is a need to develop approaches to introduce microstructures into MXene-based sensors so that their performance in sensing devices can be greatly improved.

Up to now, non-planar microstructures with different morphologies have been introduced into MXene-based piezoresistive sensors to obtain higher sensitivity and shorter response time, such as the bioinspired microspines [6], wrinkle structure [1], and protrusion structures [23]. The preparation techniques involved in these microstructures are mostly based on the use of everyday objects with surface microstructures as molds or pre-stretching methods [1,6,23]. Compared with the expensive and complex traditional photolithography technique, these micro-nano processing approaches are simple, convenient, and low-cost, but it is difficult to meet the needs of free customization. Most recently, it was demonstrated that femtosecond laser filament micro-nano processing technology can provide a useful strategy to arbitrarily adjust the morphology of the flexible substrates composing the sensors, thereby developing a robust alternative to fabricating high-performance sensors in a simple, low-cost, and fast way [27,28,29]. The constructed flexible piezoresistive pressure sensors with filament-processed microstructure exhibit high sensitivity to pressure and vibration, which can be used with success to monitor various human physiological signals [28]. Moreover, with the assistance of the principal component analysis (PCA) algorithm and artificial neural networks, the fabricated sensor can unambiguously identify different phonations, and total recognition accuracy rate can reach 93.4% [28,29]. However, from the perspective of the practical application of flexible wearable smart devices, it is necessary to further improve the sensing performance of the device to sensitively monitor both subtle and large movements of the human body.

Herein, a highly-sensitive piezoresistive sensor was assembled by combing ${\mathrm{Ti}}_{3}C_{2}T_{x}$ MXene nanosheets with honeycomb-like microarchitecture polydimethylsiloxane (PDMS) film fabricated by femtosecond laser pulses in the self-channeling regime. As stress increases, the honeycomb-like microstructures rapidly increase the connecting conductive paths between interface states so that the performance of the pressure sensor can be enhanced. The obtained MXene-based piezoresistive sensor with a honeycomb-like structure exhibits an outstanding sensitivity (0.61 kPa^−1^), a short response time (160 ms), and excellent cycling stability, and can be mass-produced based on a simple preparation process. Benefiting from the above advantages, the fabricated sensor can monitor both tiny physiological signals, such as facial expressions and artery pulse, and large human movements, such as knee bending and elbow swing. Meanwhile, it can also work effectively in distinguishing various finger gestures, providing a convenient communication tool for people with language barriers. Therefore, our work provides a competitive route for large-scale preparation of low-cost, high-sensitivity piezoresistive sensors with broad prospects in health, sports, and communication applications.

## 2. Experiment Details

**Figure 1:**
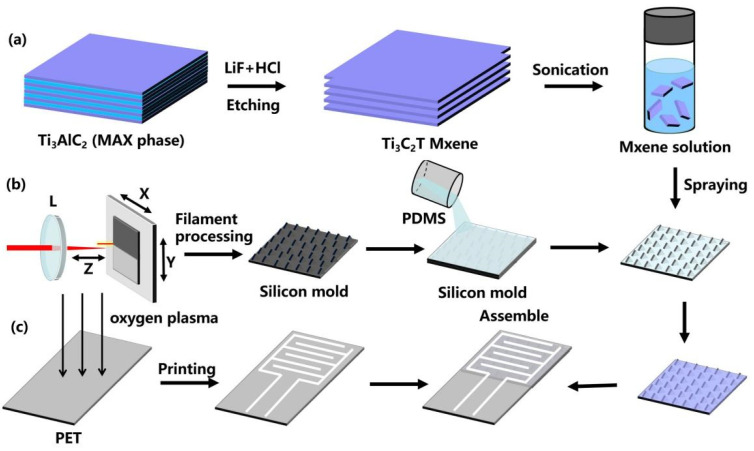
(**a**) Preparation of ${\mathrm{Ti}}_{3}C_{2}T_{x}$ MXene. (**b**) Preparation of Micro-Patterned MXene/PDMS conductive film. (**c**) Assembly of the MXene-based piezoresistive sensor.

The fabrication of the MXene-based piezoresistive sensor with a honeycomb-like structure was carried out with the procedures illustrated in Figure 1.

Preparation of ${\mathrm{Ti}}_{3}C_{2}T_{x}$ MXene. ${\mathrm{Ti}}_{3}C_{2}T_{x}$ aqueous dispersions were synthesized by chemically exfoliating the Al layer of ${\mathrm{Ti}}_{3}{\mathrm{AlC}}_{2}$ by HCl + LiF etchant, as shown in Figure 1a. First, 2 g of LiF was slowly added into 40 mL of 9 M HCl, followed by continuous stirring at room temperature for 30 min. Subsequently, 2 g of MAX phase precursor (${\mathrm{Ti}}_{3}{\mathrm{AlC}}_{2}$) powder was gradually added to the above solution for selective etching, which was stirred for 24 h at 35 °C. Then, the resulting MXene suspension was washed with deionized water and centrifuged repeatedly at 3500 rpm until the pH was about 5–6. The sediment was then dried in a vacuum oven and the resulting product was sonicated with ethanol for 1 h. Finally, the obtained powder was added into deionized water, and then sonicated and centrifuged at 3500 rpm for 3 min to collect the ${\mathrm{Ti}}_{3}C_{2}T_{x}$ supernatant. In this experiment, the concentration of the ${\mathrm{Ti}}_{3}C_{2}T_{x}$ solution was quantified to 5 mg/mL by further dilution or concentration by vacuum evaporation.

Preparation of Micro-Patterned MXene/PDMS conductive film. Figure 1b briefly shows the fabrication process of the MXene sensing thin film with a honeycomb-like structure. First, linearly-polarized laser pulses with a pulse width of 35 fs, a central wavelength of 800 nm, a repetition rate of 500 Hz, and a pulse energy of 2 mJ were generated based on a commercial Ti:sapphire laser system (Spectra-Physics, Spitfifire ACE) and a self-built optical system. After that, the laser pulse was loosely focused by a 25.4 mm-diameter fused silica lens (f = 100 cm) to form a 3 cm-long laser filament with an estimated clamped intensity of the filament core of about 150–200 TW/cm^2^. Then, the mold with the honeycomb-like microarchitecture was prepared through the interaction between the single filament and a 2 mm-thick silicon wafer. The sample translation speed and the separation between adjacent processed lines correspond to 1.25 mm/s and 100 µm, respectively. The patterned flexible PDMS thin film was then achieved by replicating the reversed micropatterns from the fabricated silicon mold. Finally, a facile and scalable drip-coating method was employed to deposit the ${\mathrm{Ti}}_{3}C_{2}T_{x}$ MXene nanosheets of high conductivity onto the microstructured PDMS thin film by van der Waals force.

Assembly of the MXene-based piezoresistive sensor. As shown in Figure 1c, the cleaned PET film was first exposed to oxygen plasma (2 min) for hydrophilic treatment. Then, the interdigitated electrode patterned silver conductive ink thin layers were printed on the PET substrate through an inkjet printer and thermally dried in an oven at 100 °C for 1 h. Subsequently, with the assistance of 3 M tape, the MXene sensing film and the interdigital electrode were assembled into the MXene-based piezoresistive sensor.

Performance Characterization. The morphology and structural analysis of the samples was performed using a field emission scanning electron microscope (SEM model Regulus8100 by Hitachi). In order to analyze the phase composition and crystal structure of the fabricated ${\mathrm{Ti}}_{3}C_{2}T_{x}$ MXene, the XRD patterns were observed by a Rigaku D/MAX 2550 diffractometer at a scan angle of 4${}^{\circ }$ to 60${}^{\circ }$. The sensing performance of the MXene-based piezoresistive sensor was measured with a homemade testing machine, which is composed of a force gauge and a data acquisition system (KeithleyDAQ6510).

## 3. Result and Discussion

**Figure 2:**
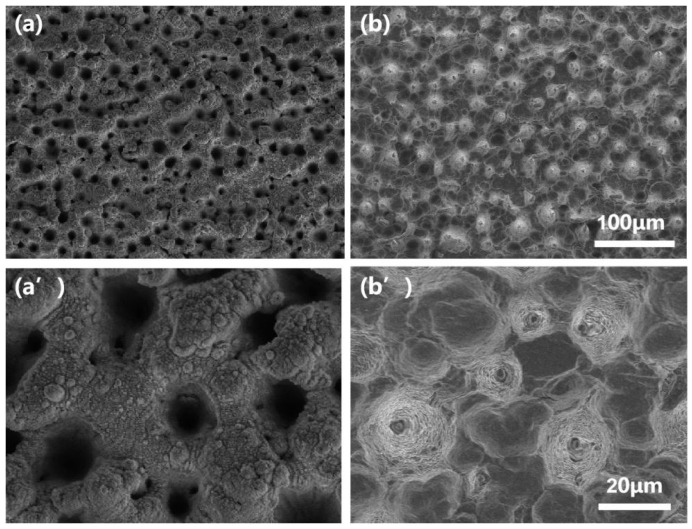
(**a**,**a’**) The top-view SEM images of the filament-processed silicon mold surface under low and high magnifications. (**b**,**b’**) Differently magnified SEM images of the fabricated MXene-based PDMS film surface.

**Figure 3:**
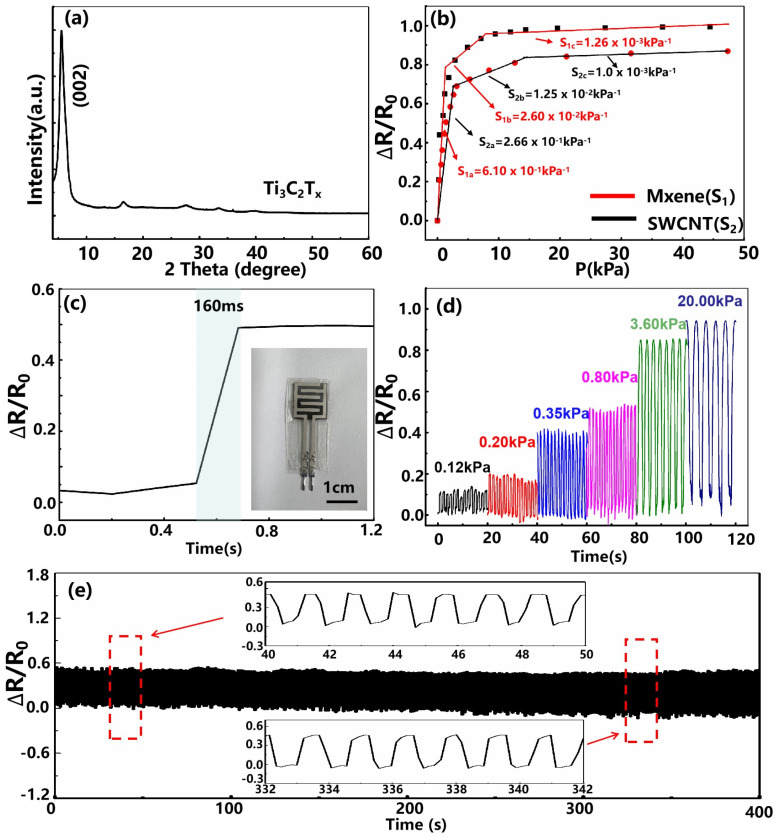
(**a**) XRD spectra of ${\mathrm{Ti}}_{3}C_{2}T_{x}$ materials. (**b**) The resistance response versus pressure change for the filament-processed MXene-based piezoresistive sensor and the SWCNTs-based piezoresistive sensor. (**c**) Instant response of the MXene-based piezoresistive sensor. Inset: Photograph of the fabricated sensing device taken by a digital camera. (**d**) Relative resistance variation of the fabricated MXene-based piezoresistive sensor under serial pressures. (**e**) Real-time resistance response of the MXene-based piezoresistive sensor for loading/unloading cycles under 0.8 kPa, at a 0.64-Hz repetition rate.

In Figure 2a,a’, we show the differently-magnified SEM images of the filament-processed silicon mold surface at the ablation speed of 1.25 mm/s, corresponding to the number of 40 laser shots hitting the same position. It can be found that highly-repeatable honeycomb-like microarchitectures with an average size of about 15–20 µm are formed on the surface after the interaction between the filament and the silicon wafer. In addition, we also conducted microscopic analysis on the surface of MXene-based PDMS film, as shown in Figure 2b,b’. From the high magnification SEM image in Figure 2b’, it can be seen that the stacked ${\mathrm{Ti}}_{3}C_{2}T_{x}$ can be densely coated on the microstructured PDMS film, which is attributed to the abundant hydrophilic groups, such as -H, -OH, generated on the surface of the MXene after chemical etching. In order to further investigate the crystalline structure of ${\mathrm{Ti}}_{3}C_{2}T_{x}$ thin film, we measured its XRD spectra, as shown in Figure 3a. The strong diffraction peak (002) located at 6${}^{\circ }$ indicates the formation of ${\mathrm{Ti}}_{3}C_{2}T_{x}$ nanosheets with hexagonal crystal structure by etching. In addition, the (104) peak related to the Al element around 39${}^{\circ }$ vanishes, revealing the successful extraction of Al-atoms from ${\mathrm{Ti}}_{3}{\mathrm{AlC}}_{2}$ and indicating that the etching degree was relatively complete during the preparation process.

Based on the above-mentioned filament-processed microstructure and MXene sensing material, we assembled and characterized the sensing performance of the MXene-based piezoresistive sensor with a honeycomb-like structure. The sensitivity, as a key parameter for evaluating device performance, can be defined as $S=(\Delta R/R_{0})/\Delta P$, where ΔR is the resistance change before and after the applied pressure, and $R_{0}$ and ΔP are the initial value without applied pressure and the change of the applied pressure. The $\Delta R/R_{0}$ versus ΔP for the MXene-based piezoresistive sensor is displayed in Figure 3, from which it can be clearly seen that the fabricated sensor shows three linear regions. The fitted sensitivities are, respectively, $6.10\times 10^{-1}$ kPa^−1^ (comparable with those reported in previous literatures [30,31]), $2.60\times 10^{-2}$ kPa^−1^, and $1.26\times 10^{-3}$ kPa^−1^, for the range of 0–1.5 kPa, 1.5–8 kPa and 8–50 kPa. For this sensor, the superior sensing performance may be attributed to the specific morphology of the filament-processed microstructure and the extraordinary properties of ${\mathrm{Ti}}_{3}C_{2}T_{x}$ nanosheets. In addition, we also compare the sensitivity and responsivity of the sensing devices constructed with different sensing materials, as shown in Figure 3b. As can be seen from Figure 3b, the MXene-based device exhibits higher responsivity, and its sensitivity under the pressure of 8 kPa is more than two times higher than that of the SWCNTs-based piezoresistive sensor (our previously prepared device) [26]. The above results indicate that the MXene-based sensor is more suitable for monitoring human activity signals and, consequently, may have the potential for health, sports, and communication applications.

The working mechanism of the MXene-based piezoresistive sensor and the existence of the three different sensitivities can be explained as follows. In the initial state, several conductive paths are formed between the MXene/PDMS conductive film and the interdigitated electrodes due to the presence of contact points. When external pressure is applied to the device, the contact area between the relatively large honeycomb-like architecture and the interdigitated electrodes increases, resulting in a significant increase in the number of conductive paths. As the pressure increases further, the parts that are already in contact provide support of a certain level, which slows down the increase rate of the conductive path. In the high-pressure regime, the honeycomb-like microstructures have been compressed to a flat shape, that is, at their maximum compression positions, and thus the increased pressure contributes only to a small increase in the conducting area of the interface state.

In addition, the fast response time of the device is also of great significance for practical applications, as it can ensure a timely response in the monitoring process of ultrafast pressure signals. As illustrated in Figure 3c, although the elastic deformation of the microstructured PDMS film will cause a certain response time delay, the fabricated device exhibits a short response time of 160 ms (less than the human body’s response time of 400 ms), which is sufficient for human activity monitoring. In order to demonstrate the fabricated sensor more intuitively, a photograph of the fabricated sensing device is shown in the inset of Figure 3c. It can be seen that the prepared sensor has an overall size of $3.0\times 1.2\;{\mathrm{cm}}^{2}$ with a thickness of 450 µm, and the sensing area occupies $1.00\times 1.25\;{\mathrm{cm}}^{2}$. Figure 3d shows the stable cyclic performance of the fabricated MXene-based piezoresistive sensor under serial pressures (multiple cyclic loading under the applied pressures of 0.12 kPa, 0.2 kPa, 0.35 kPa, 0.8 kPa, 3.6 kPa, and 20 kPa). Moreover, we also measured the long-term cyclic stability of the fabricated MXene-based piezoresistive sensor, that is, loading–unloading cycling tests at a frequency of 0.64 Hz under a pressure of 0.8 kPa (Figure 3e). It was found that under the pressure of 0.8 kPa, the relative resistance response amplitude of the device is about 50.0%, which is consistent with the obtained sensitivity curve (Figure 3b). After long-term loading and unloading tests, the shape and intensity of the electrical signal change of this sensor remains almost the same with a slight attenuation, showing its excellent long-term repeatability and stability.

**Figure 4:**
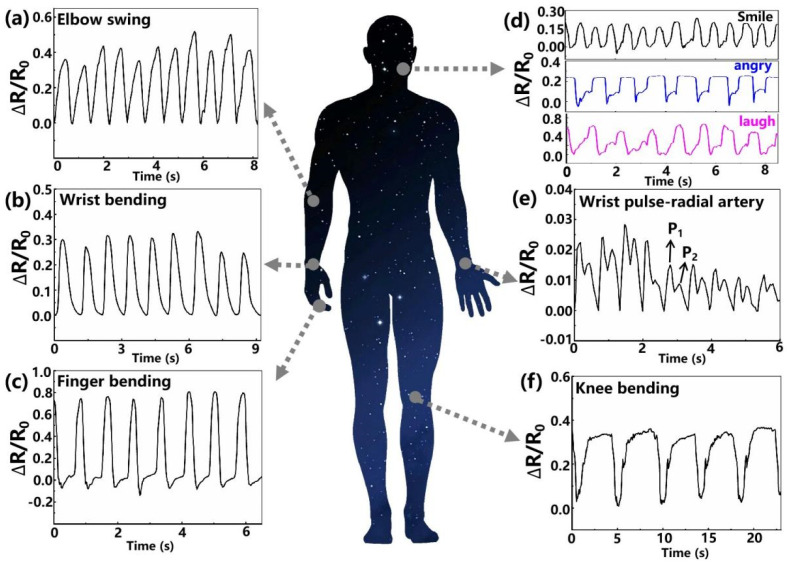
Applications of the MXene-based piezoresistive sensor for real-time monitoring of human activities. Resistance change waveforms of the wearable sensor in detecting human movements: (**a**) elbow swing, (**b**) wrist bending, (**c**) finger bending, (**d**) facial expressions, (**e**) wrist pulse, and (**f**) knee bending.

The outstanding performance of the fabricated MXene-based piezoresistive sensor with a honeycomb-like structure, with its high sensitivity, repeatability and excellent conformal contact, allows it to be useful as a wearable device for real-time tiny physiological signal and large-scale human motion monitoring. In human health monitoring, the detection of joint activities is of great significance for motion monitoring and gesture recognition. By attaching the fabricated sensor to different joint positions of the human body with the assistance of medical tape, we obtained the responsive signal waveforms for bending the elbow (Figure 4a), wrist (Figure 4b), finger (Figure 4c), and knee (Figure 4f), where each flexion of the joint maintains almost the same angle. It can be seen that the motion state can be clearly distinguished by comparing the shape and intensity of the graphs; that is, the responsivity tends to increase due to the increase of pressure during the bending process. The above results clearly demonstrate the potential of our fabricated sensor in the field of large-scale human motion monitoring.

To demonstrate the tiny deformation detection capability of our sensor, the device was attached to the subject’s cheek to monitor changes in facial muscles as expressions change. Three main emotional facial expressions, namely anger, smiling, and laughter, were investigated, as shown in Figure 4d. In fact, human facial expressions are produced by several muscle groups. It was found that when the tester makes different facial expressions, the obtained waveforms have significant differences, mainly reflected in the change of relative resistance and the movement time of micro-muscle groups. The differences in characteristic peaks are mainly attributed to the deviation of facial skin or muscle movement when people perform different expressions. It was found that when the subject makes different facial expressions, the resistance change waveforms are significantly different, which is caused by deviations in the degree of facial skin or muscle movement. From Figure 4d, the laughing expression results in the largest change in the device resistance value, which may be attributed to the fact that happy laughter requires large facial muscle fluctuations and excitation of all muscle groups on the face. In addition, when the subject repeatedly makes the same facial expression, the response curves obtained by the device show consistent characteristics.

**Figure 5:**
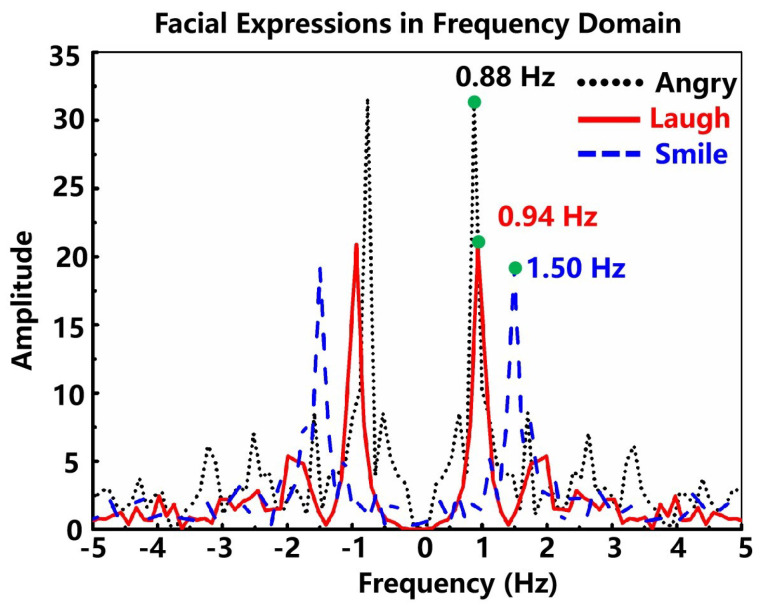
The signals of the facial expressions transformed into the frequency domain.

We have transformed the signals of the facial expressions into a frequency domain to make the difference among them more apparent, as shown in Figure 5. The main peaks of the three expressions are of different frequencies and amplitudes. The smile expression has a shorter-term muscle movement than laughter and anger, so its signal on the time domain is narrower than the other two, while it is obvious in the frequency domain that the frequency of its main peak is higher than the other two. Furthermore, though the anger and laughter are almost the same widths in the time domain, the angry expression muscle retention time is longer, which is indicated by the period of the flat top on their curves. Correspondingly, the difference between anger and laughter is much easier to detect in the frequency domain because, though the frequencies of the main peaks of the angry signal and the laughing signal are almost the same, the angry signal has higher harmonic components, which form a longer-term flat top. The facial expressions sampled with our sensor have significantly different features, so a threshold discriminant method based on signal processing or a classifier based on machine learning can be applied for pattern recognition of the expressions. Based on the aforementioned test, it is shown that our fabricated sensor can be used to recognize the delicate deformations of human facial expressions, which could contribute to observing the state of thinking of the human brain.

**Figure 6:**
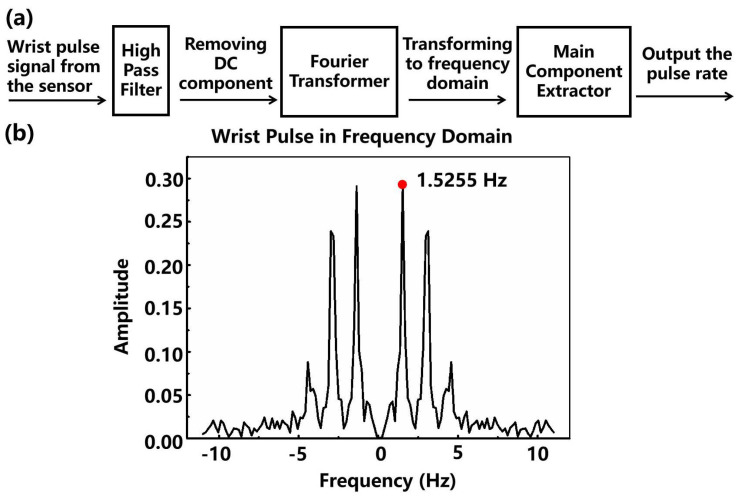
(**a**) The diagram of extracting the pulse rate from the sensor attached to the skin of the subject’s wrist. (**b**) The Fourier-transformed wrist pulse signal.

Moreover, we test the capability of the fabricated sensor to identify weak physiological signals. The sensor was tightly attached to the skin of one’s wrist for non-invasive continuous monitoring of the radial artery blood pressure wave. As shown in Figure 4e, the obtained pulse signal is consistent with the typical radial artery characteristic waveform, containing two distinct peaks, namely, the systolic (P1) and diastolic (P2) peaks [32]. The signal processing system matched with the sensor to automatically extract the pulse rate is shown in Figure 6a. Firstly, a high pass filter with a cut-off frequency of 0.5 Hz is applied to remove the direct-current component. Then, a Fourier transformer module transforms the time-domain radial artery pulse signal to the frequency domain. After that, the main component extractor picks up the most significant frequency component, which indicates the frequency of the artery pulsing. As shown in Figure 6b, the most significant frequency component is at 1.5255 Hz, equivalent to 91.5 bpm, close to the actual pulse rate of the subject, 90 bpm. Since the commercial wearable bracelets allow measurement error within ±5 bpm in the resting state, our sensor is qualified for commercial applications. Additionally, some important medical information for health monitoring can also be obtained, such as the reflection index (R.I.) and the stiffness index (S.I.).

**Figure 7:**
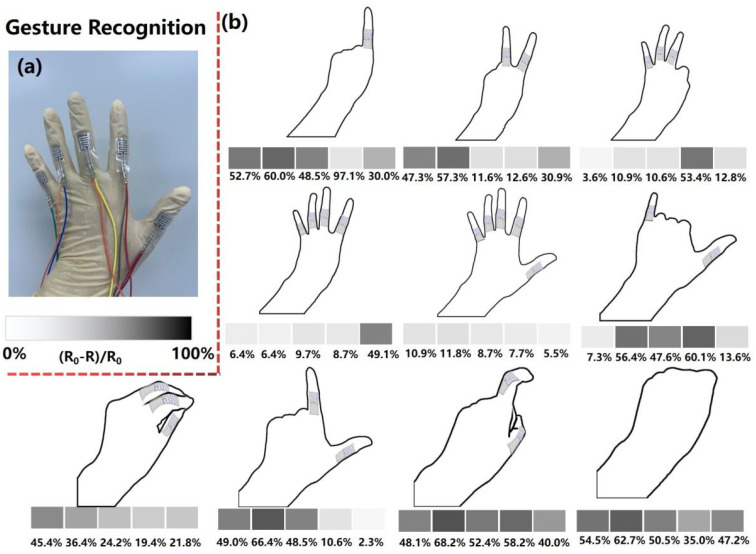
(**a**) The gesture recognition glove based on the fabricated piezoresistive sensor. The prepared five MXene-based piezoresistive sensing devices were assembled at the joints of the glove fingers. (**b**) Gestures of Arabic numerals and their corresponding responsive output.

Furthermore, the applications of the fabricated MXene-based piezoresistive sensor for exquisite and sophisticated finger gesture recognition are illustrated in Figure 7. As shown in Figure 7a, the gesture recognition glove is assembled by integrating the smart glove with our fabricated MXene-based piezoresistive sensor. Five independent sensing devices are stuck on the joint parts of the glove fingers to measure the bending and tilting angles of the five fingers. Figure 7b shows the schematic diagrams of Arabic numeral gestures in a daily scenario and the corresponding responsive output obtained. It was found that the numbers “0” to “9” can be unambiguously distinguished according to the intensity of the piezoresistive output, which mainly depends on the bending angle and number of the finger joints. From number “1” to number “5”, the highly responsive piezoresistive output gradually decreases due to the reduction in the number of bent fingers. When the gesture matches the number “6”, the index, middle, and ring fingers curl up, resulting in a reduction in the resistance of the corresponding sensors. When the gesture transitioned from the number “7” to the number “0”, the amplitude of the piezoresistive response elevated gradually, which may be attributed to the increase in the magnitude and number of finger bends. Thus, our MXene-based sensor with a honeycomb-like structure can be used to register various sophisticated gestures, which has broad application prospects in sign-to-speech translation, virtual reality, and other fields.

## 4. Conclusions

In summary, we fabricated a sensitive flexible MXene-based piezoresistive sensor with a honeycomb-like structure through drip-coating MXene nanosheets combined with femtosecond filamentating pulses processing. With the specific morphology of the filament-processed microstructure and the extraordinary properties of ${\mathrm{Ti}}_{3}C_{2}T_{x}$ nanosheets, the obtained MXene composite sensor features a high sensitivity of 0.61 kPa^−1^, which is more than two times higher than that of our previously prepared SWCNTs-based piezoresistive sensing device [26]. Additionally, the device also demonstrates a short response time of 160 ms and excellent repeatability under loading/unloading cycles. The sensing mechanism of the fabricated device can be explained by the change in the conducting area of the interface state between the MXene/PDMS conductive film and the interdigitated electrodes. This extraordinary sensing performance attributes enabled us to realize the monitoring of sensing signals from tiny muscle movement to large-scale human motions, including artery pulse, facial expressions, and joint motion. Subsequently, we assembled a smart glove that was used with success for exquisite and sophisticated gesture recognition. Therefore, we hope that our fabricated sensing device can be used in next-generation wearable electronic devices for healthcare monitoring and human activity real-time detection.

## Data Availability

Not applicable.
